# Use of Elamipretide in patients assigned treatment in the compassionate use program: Case series in pediatric patients with rare orphan diseases

**DOI:** 10.1002/jmd2.12335

**Published:** 2022-09-21

**Authors:** Mary Kay Koenig, Sam Nick Russo, Kim L. McBride, Hans Tomas Bjornsson, Brynja Bjork Gunnarsdottir, Amy Goldstein, Scott A. Falk

**Affiliations:** ^1^ The University of Texas McGovern Medical School, Center for the Treatment of Pediatric Neurodegenerative Disease Houston Texas USA; ^2^ Division of Genetic and Genomic Medicine and the Heart Center Nationwide Children's Hospital, Department of Pediatrics College of Medicine Ohio State University Columbus Ohio USA; ^3^ Landspitali University Hospital Reykjavik Iceland; ^4^ Faculty of Medicine University of Iceland Reykjavik Iceland; ^5^ Mckusick‐Nathans Department of Genetic Medicine Johns Hopkins University Baltimore Maryland USA; ^6^ Children's Hospital of Philadelphia Philadelphia Pennsylvania USA; ^7^ Perelman School of Medicine of the University of Pennsylvania Philadelphia Pennsylvania USA

**Keywords:** Barth syndrome, Cardiolipin, Elamipretide, MEGDEL, mitochondrial disease, Sengers syndrome

## Abstract

Several mitochondrial diseases are caused by pathogenic variants that impair membrane phospholipid remodeling, with no FDA‐approved therapies. Elamipretide targets the inner mitochondrial membrane where it binds to cardiolipin, resulting in improved membrane stability, cellular respiration, and ATP production. In clinical trials, elamipretide produced clinical and functional improvements in adults and adolescents with mitochondrial disorders, such as primary mitochondrial myopathy and Barth syndrome; however, experience in younger patients is limited and to our knowledge, these are the first case reports on the safety and efficacy of elamipretide treatment in children under 12 years of age. We describe the use of elamipretide in patients with mitochondrial disorders to provide dosing parameters in patients aged <12 years.

**Synopsis**

Dosing parameters for the use of elamipretide in patients <12 years of age with mitochondrial diseases caused by pathogenic variants that impair membrane phospholipid remodeling are provided.

## 1 BACKGROUND

Several mitochondrial diseases are caused by pathogenic variants that impair membrane phospholipid remodeling, including Barth syndrome (MIM #302060), 3‐methylglutaconic aciduria, deafness, encephalopathy, Leigh‐like disease (MEGDEL, MIM #614739), and Sengers syndrome (MIM #212350). Although there are no US Food and Drug Administration (FDA)–approved therapies for these conditions, the FDA has provided guidance on expanded access for the use of investigational drugs, including elamipretide, for patients with serious diseases who lack therapeutic alternatives when there is a biologic plausibility to predict efficacy.^1^ Elamipretide, a cell‐permeable peptide that has been shown to target the inner mitochondrial membrane where it binds to cardiolipin, resulting in improved membrane stability, cellular respiration, and ATP production,^2^, ^3^ has been evaluated in adult and adolescent patients with mitochondrial disorders, such as primary mitochondrial myopathy and Barth syndrome, producing clinical and functional improvements.^4^, ^5^, ^6^ There is little experience with elamipretide in younger patients and those with other mitochondrial diseases. We describe the use of elamipretide in a series of patients with various mitochondrial disorders with a goal of providing insight on dosing and rationale for its use.

### 1.1 Case series

#### 1.1.1 Case 1: Barth syndrome

A male patient presenting with heart gallop within hours of birth was found to have lactic acidosis and severe left ventricular (LV) dysfunction characterized by severe dilated cardiomyopathy and an ejection fraction (EF) of approximately 20%. The cardiomyopathy was unresponsive to medical management. Exome sequencing identified a pathogenic variant in Tafazzin (*TAZ* c.212C > T), consistent with a diagnosis of Barth syndrome (diagnosis based on entry in a previous study at 3 weeks of age that included rapid genome sequencing) and related cardiomyopathy. It was decided to initiate elamipretide at 3 weeks of age as his EF (Table 1) had not significantly improved on maximal medical therapy that included milrinone, sacubitril/valsartan, and carvedilol. The initial dose of elamipretide was 0.25 mg/kg/day administered intravenously with weight‐based upward titration to 0.5 mg/kg/day beginning at 1 week. Each dose was administered over 2 h. At 4 weeks, the patient was switched to subcutaneous (SQ) dosing at 0.5 mg/kg/day (10 mg/ml formulation of elamipretide) and discharged from the hospital on that dose. SQ dosing was chosen because of the concerns for a high infection risk with an indwelling central catheter (PICC line) in the setting of neutropenia, a feature of Barth syndrome. An IND provided the approval. Additional medications administered since discharge included filgrastim, sacubitril/valsartan, carvedilol, and spironolactone. Since Barth syndrome is often associated with reduced arginine levels, arginine was also added to the medication regimen. This dosing schema resulted in a gradual improvement of EF to between 45% and 55%, although dyskinesia of the LV remained (Table 1). Neutropenia was improved on filgrastim, with normal absolute neutrophil counts ranging from 1020 to 1200 mm^3^ after therapy was started. The comprehensive metabolic panel and CK were normal after 1 month of age, although there was a mild persistent lactic acidosis ranging from 2.0 to 4.4 mmol/L. At 4 months of age, the patient was meeting all developmental milestones. There were no adverse events related to elamipretide, regardless of the route of administration (IV or SQ). At 5.5 months of age, the patient died with autopsy listing the cause of death as undetermined with other significant conditions: Barth syndrome, unsafe sleep environment, and *Klebsiella pneumoniae* bacteremia. The total time on treatment with elamipretide was 150 days.

**TABLE 1:** Barth syndrome patient (Case 1) imaging data

Date	Modality	SF%	EF%	LVEDD mm (Z)	LV hypertrophy	LV function	RV function
11/12/20	TTE	13	26–39	19 (−0.1)		Moderate‐severe	Normal
11/13/20	TTE	21	44–48	19 (0)		Mild, dyskinetic	Low‐normal to mild dysfxn
11/14/20	TTE	26			Severe	Mild, dyskinetic	Mild
11/15/20	TTE	19	45	20 (+0.8)		Mild, dyskinetic	Mild
11/19/20	TTE	10	40–50	21 (+1.1)		Moderate, dyskinetic	Mild
11/24/20	TTE	17	25–28	23 (+2.6)	Moderate	Severe, dyskinetic	Normal
11/25/20	TTE	11	35	21 (+1.2)	Moderate	Severe, dyskinetic	Normal
11/30/20	TTE	13	26–32	22 (+1.8)	Moderate	Severe, dyskinetic	Normal
*12/02/20 Initiation of sacubitril‐valsartan*							
12/4/20	TTE	18	37–38	20 (+0.7)	Moderate	Moderate, dyskinetic	Low‐normal
*12/05/20 Initiation of elamipretide at 0.25 mg/kg*							
12/7/20	TTE	15	39–66	20 (−0.3)	Mild	Low‐normal	Normal
*12/10/20 Off milrinone last*							
*12/12/20 Elamipretide to 0.5 mg/kg*							
12/12/20	TTE	22	41–53	18 (−1.0)	Mild	Mild‐moderate, dyskinetic	Normal
12/16/20	TTE		41–54	20 (+0.2)	Mild	Mild, dyskinetic	Normal
12/22/20	TTE	29	48	21 (+0.3)	Mild	Mild, dyskinetic	Normal
12/30/20	TTE	23	51–54	20 (−0.3)	Mild, LVNC	Mild, dyskinetic	Normal
1/15/21	TTE	21	44–46	22 (−0.1)	Mild, LVNC	Mild, dyskinetic	Normal
2/19/21	TTE	22	58	19 (−2.4)	Mild, LVNC	Mild, dyskinetic	Normal
4/15/2021	TTE	30	50–57	23 (−0.8)	Mild, LVNC	Low‐normal, dyskinesic	Normal

Abbreviations: EF, ejection fraction; LVEDD, left ventricle end‐diastolic dimension; LVNC, left ventricular noncompaction; RV, right ventricle; SF, shortening fraction; TTE, transthoracic echocardiogram.

#### 1.1.2 Case 2: MEGDEL syndrome

**FIGURE 1:**
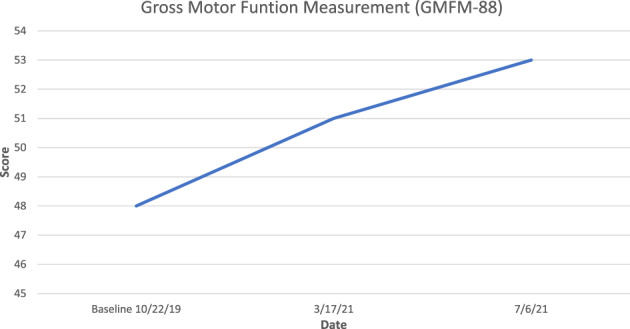
Gross motor function measurement at baseline (prior to elamipretide dose) and during elamipretide treatment (Case 2)

A male patient presented to the clinic with MEGDEL syndrome secondary to compound heterozygous pathogenic variants in the *SERAC1* gene (c.548G > A and c.222_228dupAT). The patient had experienced significant developmental delays in early motor development (i.e., not sitting alone until 14 months or crawling until 20 months and ambulating with a walker at 3 years). Dysarthria resulted in impediments to communication that caused him to be difficult to understand. At his best, he spoke in 4‐to‐5‐word sentences, and his speech was approximately 50% understandable to a stranger. Although he experienced sporadic brief regressions with illness, he mostly gained skills until the age of 5 years when he began to display regression, likely due to myopathy. After this age, he began to lose the ability to use his walker and by age 6 could no longer stand. Articulation also became progressively more dysarthric. An echocardiogram and renal evaluations at age 4 revealed no abnormalities. At 3 years of age, the patient experienced an acute episode in which he was found unresponsive and had a lactic acid level of 7.9 mmol/L. He received IV fluids, and symptoms resolved after an overnight hospitalization. The symptoms have not recurred. At 6 years and 7 months of age, elamipretide was initiated at a SQ dose of 10 mg/day (0.58 mg/kg/day) with a predetermined plan to increase the dose to 20 mg/day when he reached a weight of 20 kg. Since this weight has not been attained, the patient remains on 10 mg SQ daily at 7 years and 10 months. The patient experienced mild injection site reactions, but otherwise no adverse reactions to elamipretide were observed. After 1 year and 3 months of therapy at this dose, the patient has demonstrated developmental progress and a lack of continued regression (Table 2 and Figure 1). While the patient's parents noted improved stamina and strength, his push frequency and one‐push stroke on the wheelchair propulsion test significantly improved. He is again speaking in 3‐to‐4‐word sentences, and speech is more understandable, most likely due to improved motor function. He is able to sit alone, stand with support, and use his gait trainer.

**TABLE 2:** MEDGEL syndrome patient (Case 2) wheelchair propulsion test data

Date	Speed (m/s)	Push frequency (cycle/s)	Effectiveness (m/cycle)	Time forward wheeling (s)	Ramp Ascent (s)	One‐stroke push (in)
10/22/19 (prior to elamipretide treatment)	0.31	0.375	0.83	79.29	79.37	24
7/6/21 (during elamipretide treatment)	0.22	0.545	0.83	<60	68.40	37

#### 1.1.3 Case 3: Sengers syndrome

A male patient presented at 3 weeks of age with severe hypertrophic cardiomyopathy, bilateral cataracts, and significant hypotonia. Physical examination and whole genome analysis revealed he was homozygous for an Icelandic founder mutation (p.Ile348AsnfsTer39) in the acylglycerol kinase (*AGK*) gene, and a diagnosis of Sengers syndrome was made based on symptoms and genotype. Genetic analysis also revealed that he was a carrier of two variants in the *GPT2* gene, one from his mother [c.247C > T p(Arg92*)] and the other from his father [c.371G > C p(Ser124ThrI)], the latter being a variant of uncertain significance; however, his phenotype was not consistent with *GPT2*‐deficiency, so this diagnosis was disregarded. It is worth noting that his presentation was unusually aggressive compared with other Icelandic patients carrying these particular founder variants, so it is not impossible that the other variants affected the phenotype in some way. The boy was initiated on beta‐blocker therapy that was gradually titrated up (including during the trial with elamipretide), and beginning at age 3 months, he received elamipretide (0.25 mg/kg IV infused over 2 h [per protocol], every 24 h), and at 4 months of age, it was increased to 0.5 mg/kg IV infused over 2 h every 24 h. This dose was then increased per weight but not otherwise modified. Therapy with elamipretide continued for approximately 6 months. The child was also treated with carnitine and coenzyme Q10 supplements by a neurologist. The total time on treatment with elamipretide for this patient was 187 days.

**FIGURE 2:**
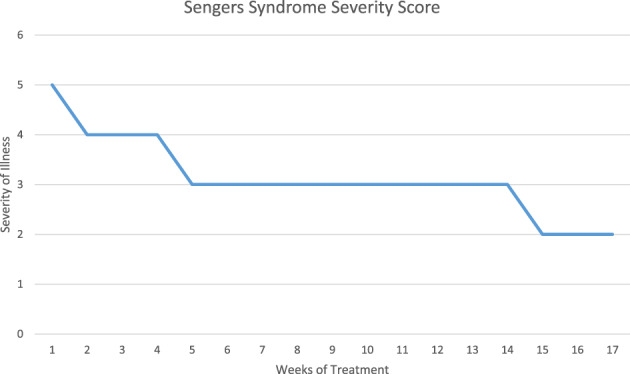
Severity of illness score during the first 4 months of treatment with elamipretide (Case 3)

Pharmacokinetic (PK) analysis, including area under the plasma concentration‐time curve (AUC) and peak plasma concentration, showed that elamipretide exposure was similar to that observed in other elamipretide trials.^7^ Following the initiation of therapy, the patient experienced subjective improvements from the prior week's evaluation in 13 of 21 visits using a Clinical Global Impression scale with the severity of illness (global) score improving from “markedly ill” to “borderline ill” during treatment (Figure 2). Cardiac improvements included an increase in the LV end‐diastolic internal dimension (Z‐score from −2.7 to +0.7), a decrease ventricular septal thickness (Z‐score from 4.7 to 3.5), and a decrease in LV posterior wall thickness (Z‐score from 5.8 to 4.7). There were no side effects that were attributable to elamipretide. The patient experienced intermittent lactic acidosis and neutropenia that were considered secondary to his condition. After 6 months of treatment, the patient underwent an elective PEG (percutaneous endoscopic gastrostomy) placement after which he experienced cardiac decompensation, followed by multiorgan failure and death.

## 2 DISCUSSION

These cases describing the expanded access use of elamipretide in patients with a range of diseases associated with mitochondrial dysfunction provide insight regarding elamipretide dosing, especially dosing in young patients (Table 3). The patients described share several phenotypic traits related to mitochondrial dysfunction. Barth syndrome is an X‐linked genetic disease caused by mutations in *TAZ*, the gene that encodes for a transacylase involved in the remodeling of cardiolipin. Cardiolipin is a crucial phospholipid found in the inner membrane of mitochondria that is necessary for the proper functioning of the respiratory chain.^8^ Barth syndrome is characterized by cardiomyopathy, neutropenia, hypotonia, and growth abnormalities.^9^, ^10^ MEGDEL is caused by biallelic pathogenic variants in the *SERAC1* gene that produces mitochondrial dysfunction.^11^ Alterations in the gene produce changes in the acyl chain composition of cardiolipin, resulting in abnormal oxidative phosphorylation.^11^ Sengers syndrome is a mitochondrial disease caused by mutations in mitochondrial *AGK*, which is required for the structural integrity of mitochondria and contributes to the synthesis of cardiolipin. The disease is characterized by hypertrophic cardiomyopathy, skeletal myopathy, and cataracts.^12^, ^13^ Elamipretide 40 mg/day has been evaluated in patients aged ≥12 years with Barth syndrome and in those aged ≥16 years with primary mitochondrial myopathy,^4^, ^5^, ^6^ but there is very limited information on dosing for younger patients and those with other mitochondrial disorders. In healthy adults and patients with primary mitochondrial myopathy, IV and SQ doses of elamipretide up to 0.25 mg/kg/h (up to 140 mg/day) for up to 7 days have been used in clinical investigations.^7^ PK analyses suggest that elamipretide shows dose‐proportional exposure as measured by area under the plasma concentration‐time curve (AUC) and maximum plasma concentrations at doses ranging from 2 to 80 mg^7^. In adults, there was no apparent effect of age on the PKs of elamipretide, although since elamipretide is eliminated renally, elamipretide exposure increases significantly as renal function decreases. In these instances, elamipretide was well tolerated with few systemic adverse events reported, primarily consisting of headache and dizziness.^4^, ^5^, ^6^ The most common adverse events are local injection‐site reactions. In our case series, dosing in younger patients assumed that the exposure‐response relationship would be similar to that in adolescents/adults. Therefore, the target dose of elamipretide for young patients was established by estimating the dose that would demonstrate comparable PK exposure (i.e., AUC) to that seen with a 40 mg dose in adults. Physiological‐based PK modeling of pediatric physiology and its impact on the PKs of elamipretide were used to determine the dose. The results indicated that weight‐normalized clearance in relation to blood flow does not differ significantly across the ages simulated. Therefore, plasma concentration‐time profiles and AUC were expected to be similar for pediatric patients <12 years of age when utilizing weight‐normalized doses. Based on our experience, a dose of approximately 0.5 mg/kg/day is well tolerated in pediatric patients <12 years of age. Alternatively, weight‐based doses of 5, 10, and 20 mg/day for patients weighing <10, 10–20, and 20–40 kg, respectively, appear to be reasonable. Given the small number of patients in this series, some weight‐based dosing recommendations were extrapolated from our findings, and therefore no safety data exists for those categories.

**TABLE 3:** Elamipretide dosing recommendations

Age (years)	Weight range (kg)	Daily dose
<1^a^	N/A	This SQ product not to be used for patients under 1 year of age
≥1	<10^b^	5 mg SQ daily
	10–20	10 mg SQ daily
	20–40^b^	20 mg SQ daily
	>40^b^	40 mg SQ daily

^a^ All patients under 1 year of age should receive intravenous elamipretide. Do not administer subcutaneous (SQ) elamipretide product via the intravenous route. Contact EAP administrator for further information.

^b^ Our case studies did not include patients in these weight ranges and therefore the data is theoretical and based on extrapolation of the presented findings.

## 3 CONCLUSIONS

These cases demonstrate that weight‐based dosing of elamipretide is well tolerated in patients <12 years of age. This experience may guide the administration of elamipretide for future patients enrolled in the expanded access program.

## AUTHOR CONTRIBUTIONS

All authors participated in the data collection, data interpretation, and writing of these case reports. All authors had final responsibility for the decision to submit for publication.

## FUNDING INFORMATION

The authors wish to thank the staff at Stealth BioTherapeutics for providing drugs through their Expanded Access Program, and particularly the efforts and support of Anthony Abbruscato, Pharm D, and Donna Cowan, CCRC.

## CONFLICT OF INTEREST

Hans Tomas Bjornsson is on a patent describing Elamipretide for Sengers syndrome. All other authors declare no conflict of interest.

## ETHICS STATEMENT

Elamipretide injection and Elamipretide Delivery System are intended for use only as investigational products and should be used exclusively by selected Investigators who are experienced with conducting clinical research and in accordance with investigational protocols approved by an Institutional Review Board/ Ethical Committee. All patients provided their informed consent to participate in the Elamipretide EAP and to have their case information made public in accordance with the Privacy Rule standards established by HIPAA. All procedures followed were in accordance with the ethical standards of the responsible committee on human experimentation (institutional and national) and with the Helsinki Declaration of 1975, as revised in 2000. Informed consent was obtained from all patients for being included in the study.

## Data Availability

Due to privacy concerns for individual patients the source data cannot be made openly available but is held in a repository by the contributing authors.
